# Integrated Relaxation Pressure Classification and Probe Positioning Failure Detection in High-Resolution Esophageal Manometry Using Machine Learning

**DOI:** 10.3390/s22010253

**Published:** 2021-12-30

**Authors:** Zoltan Czako, Teodora Surdea-Blaga, Gheorghe Sebestyen, Anca Hangan, Dan Lucian Dumitrascu, Liliana David, Giuseppe Chiarioni, Edoardo Savarino, Stefan Lucian Popa

**Affiliations:** 1 Computer Science Department, Technical University of Cluj-Napoca, 400027 Cluj-Napoca, Romania; zoltan.czako@cs.utcluj.ro (Z.C.); gheorghe.sebestyen@cs.utcluj.ro (G.S.); anca.hangan@cs.utcluj.ro (A.H.); 2 Second Medical Department, “Iuliu Hatieganu” University of Medicine and Pharmacy, 400027 Cluj-Napoca, Romania; ddumitrascu@umfcluj.ro (D.L.D.); Liliana.david@umfcluj.ro (L.D.); popa.stefan@umfcluj.ro (S.L.P.); 3 Division of Gastroenterology, University of Verona, AOUI Verona, 37134 Verona, Italy; chiarioni@alice.it; 4 Gastroenterology Unit, Department of Surgery, Oncology and Gastroenterology, University of Padua, 35100 Padova, Italy; edoardo.savarino@unipd.it

**Keywords:** machine learning, convolutional neural network, high-resolution esophageal manometry, integrated relaxation pressure

## Abstract

High-resolution esophageal manometry is used for the study of esophageal motility disorders, with the help of catheters with up to 36 sensors. Color pressure topography plots are generated and analyzed and using the Chicago algorithm a final diagnosis is established. One of the main parameters in this algorithm is integrated relaxation pressure (IRP). The procedure is time consuming. Our aim was to firstly develop a machine learning based solution to detect probe positioning failure and to create a classifier to automatically determine whether the IRP is in the normal range or higher than the cut-off, based solely on the raw images. The first step was the preprocessing of the images, by finding the region of interest—the exact moment of swallowing. Afterwards, the images were resized and rescaled, so they could be used as input for deep learning models. We used the InceptionV3 deep learning model to classify the images as correct or failure in catheter positioning and to determine the exact class of the IRP. The accuracy of the trained convolutional neural networks was above 90% for both problems. This work is just the first step in fully automating the Chicago Classification, reducing human intervention.

## 1. Introduction

The esophagus is a muscular tube that extends from the bottom part of the throat (called the hypopharynx) to the stomach. The main function of the esophagus is to transport solids and liquids into the stomach. The complex synchronization of esophageal striated and smooth muscles allows food propagation. Problems emerge when patients have difficulty swallowing or in cases of gastric reflux. These disorders can be investigated using techniques such as an upper gastrointestinal swallow study, esophagogastroduodenoscopy, pH monitoring, and esophageal manometry. In this work we will focus on esophageal motility disorders (EMD) and their diagnosis using high-resolution esophageal manometry (HRM). Esophageal HRM uses solid state or water perfused catheters, with as many as 36 circumferential sensors which generate color pressure topography plots, representing the pressures generated in the esophageal body secondary to muscle contractions during a swallow [1,2,3,4].

**Figure 1:**
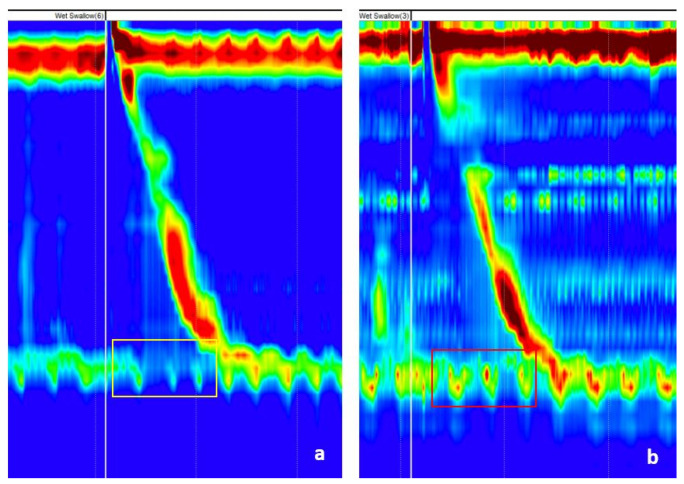
(**a**) Normal vs. (**b**) high integrated relaxation pressure (IRP). The IRP is measured at the level of the lower esophageal sphincter, in the first ten seconds after the beginning of a swallow (the region of interest is marked with a yellow/red rectangle).

Efficient esophageal transport necessitates a coordinated, sequential motility pattern that pushes food from above while clearing acid and bile reflux from below. Disruption of this highly integrated muscular action limits food and fluid delivery while also causing an unpleasant sensation of dysphagia and chest discomfort. Peristalsis is a movement pattern produced by the synchronization of these concurrently contracting muscle layers. Peristalsis is a synchronized, consecutive contraction wave that spans the length of the esophagus, pushing food to the stomach. In gastroenterology, esophageal motility problems are not rare. The spectrum of these disorders ranges from achalasia and esophago-gastric junction outflow obstruction to minor disorders of peristalsis. Motility patterns during swallows give important information regarding esophageal contractility and sphincter relaxation in response to food administration. Esophageal manometry is the measurement of esophageal movement and pressure. Conventional esophageal manometry measures contraction and pressure using probes positioned at every 5 cm in the esophagus. This technique has recently progressed, and HRM has replaced conventional esophageal manometry as the gold standard. HRM transmits intraluminal pressure data through a high-resolution catheter, which is then translated into dynamic esophageal pressure topography graphs (see example in Figure 1). An esophageal motility problem can be diagnosed by measuring the integrated relaxation pressure (IRP) and contractile function. According to the Chicago Classification algorithm [1,2,3], the IRP is the first parameter to be evaluated because it differentiates between disorders of esophago-gastric junction outflow and disorders of peristalsis. IRP measures the “opening” of the lower esophageal sphincter (LES) during swallowing, and represents the average lowest pressure through the esophago-gastric junction (which includes LES) for four contiguous or non-contiguous seconds, from a 10-s window following deglutitive upper esophageal sphincter relaxation [3]. A high IRP suggests that there is a disorder of the esophago-gastric junction outflow, while a normal IRP would point to a disorder of peristalsis. This is the reason why one of the objectives of this paper was to automatically determine if the IRP is normal or higher than the cut-off. Examples of normal and higher than cut-off IRPs are presented in Figure 1. Because the final diagnosis is made manually by trained physicians, the positioning of the catheter can influence the decision of the specialist. For example, the IRP could be sensitive to wall-catheter contact or catheter movement. In addition, detecting catheter positioning failure is critical, because in cases of failure, the manometry recordings cannot be interpreted. Furthermore, the reliance on subjective experience can also lead to inaccurate diagnosis. Machine learning, on the other hand, might give a feasible solution to HRM’s subjective interpretation concerns. A huge amount of raw manometry data combined with deep learning models might be used to identify the unique patterns that distinguish the different phenotypes of EMD. They could be synthesized or encoded as novel outcomes/features that might possibly generalize better than pre-defined features based on restricted datasets. 

The objective of our current research was to prepare the input data that will be used for the Chicago Classification. There are two steps involved in preparing the data, which we wanted to automatize:

1. To filter out the images for which the input probe was not correctly positioned.
2. To determine the IRP parameter for the correct images.

To achieve these objectives, we devised a machine learning based solution for detecting probe positioning failures in HRM images, which can be used before applying the Chicago Classification algorithm. In this way the precision of the EMD diagnosis is maximized. Furthermore, a classifier to automatically determine whether the IRP is normal or higher than the cut-off, based solely on the raw pressure topography images, was created. As mentioned above, determining the IRP type is one of the most important steps in the Chicago algorithm, so this work is the first step towards automating the Chicago Classification algorithm using machine learning techniques. Automating this algorithm could reduce the costs, because the EMD diagnosis would be automatically made, requiring a nurse to position the catheter, with the minimal intervention of a physician. The paper is organized as follows: in Section 2 the solution used to create the classification pipeline is described, in Section 3 some experimental results are presented, in Section 4 similar solutions are described, while Section 5 concludes the research.

## 2. Material and Methods

### 2.1. Raw Data Analysis

All records of esophageal HRM from our database (October 2014–February 2021) were reviewed. These records were from patients referred to our department from all parts of Transylvania, Romania, for this investigation. Most of the patients had complained of esophageal symptoms, such as dysphagia, chest pain, or heartburn. Our center is a reference center for diagnosing achalasia; therefore, almost half of the patients had achalasia. The manometry was performed after at least 6 h of fasting, using the ISOLAB manometry system (Standard Instruments GmbH, Karlsruhe, Germany) and a solid-state catheter with 36 sensors (Unisensor^®,^, Zurich, Switzerland). The catheter was inserted trans-nasally and positioned with at least 3 sensors in the stomach. The protocol for the examination consisted of a baseline recording of 2 min, followed by 10 wet 5 mL swallows, spaced at more than 30 s, with the patient in the supine position and the thorax angulated at 30°. Every wet swallow was marked during the exam, by the performing nurse or physician, with a white vertical line from the software. In this way, it was ascertained that only wet swallows (also called test swallows) would be analyzed, while all other swallows (dry swallows) would be ignored, based on current recommendations. In our study, the upper normal limit of IRP, using an Unisensor^®^ probe, was set at 28 mmHg [3].

Previous studies showed that the diagnostic accuracy of HRM for EMD is influenced by the interpreter’s experience [5,6]. Overall inter-observer agreement was “moderate” (kappa 0.51), and “substantial” (kappa > 0.7) for type I and type II achalasia [6]. For other disorders of peristalsis with normal relaxation of the LES, the agreement was even lower [5,6]. Given these observations, the datasets were prepared and labeled by two experts from the second Medical Department from Cluj-Napoca, Romania in collaboration with a specialist from the Division of Gastroenterology of the University of Padua and an expert from the Division of Gastroenterology of the University of Verona, Italy. In cases of disagreement between observers, the images were discussed, and a consensus was reached. 

**Figure 2:**
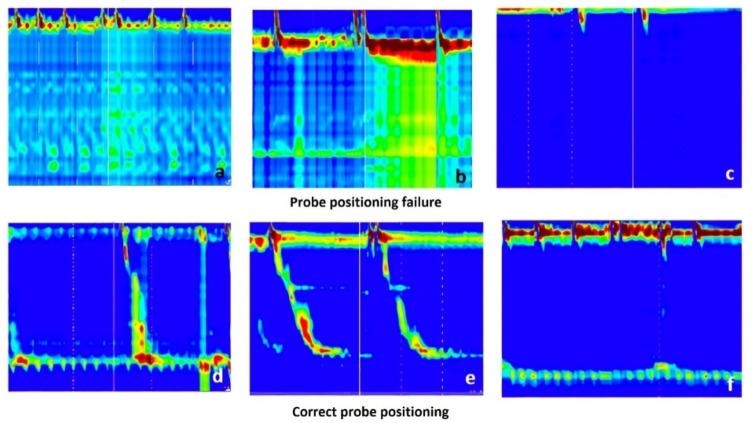
Failure in probe positioning vs. correct probe positioning; (**a**) Lower esophageal sphincter (LES) is not clearly visible. There are several zones of high pressure, in the lower part of the esophagus, but with “mirror” effect suggesting a folded catheter; (**b**) LES is visible, however, during a swallow with pressurization, there is also an important pressurization at the gastric level, which is a “mirror” effect, suggesting that the probe coiled in the esophagus; (**c**) LES is not visible at all. In the lower images (**d**–**f**), the LES is clearly visible (as a green-yellow line in the lower part of the image, determined by the pressure of the LES), and below LES there is a blue band, suggesting that some sensors are in the stomach (the gastric pressure is the reference for manometric measurements).

The first dataset for detecting the probe positioning failure contained a total of 2437 raw images from which 67 images were for positioning failure (see Figure 2) and 2370 images were for correct probe positioning (Figure 2). The mean age of the patients was 48.5 ± 16.3 years old, and 55% were males. In addition, 20% of patients had a normal esophageal HRM, 45.7% had achalasia, and the remaining were classified as follows: 13.3% ineffective esophageal motility, 7.4% absent contractility, 6.6% esophago-gastric junction outflow obstruction, 2.0% jackhammer esophagus, 1.6% fragmented peristalsis, and 0.4% distal esophageal spasm. The probe could not be placed in 8 patients (3.1%), and those images were excluded from the second dataset, and thus from further analysis. 

**Figure 3:**
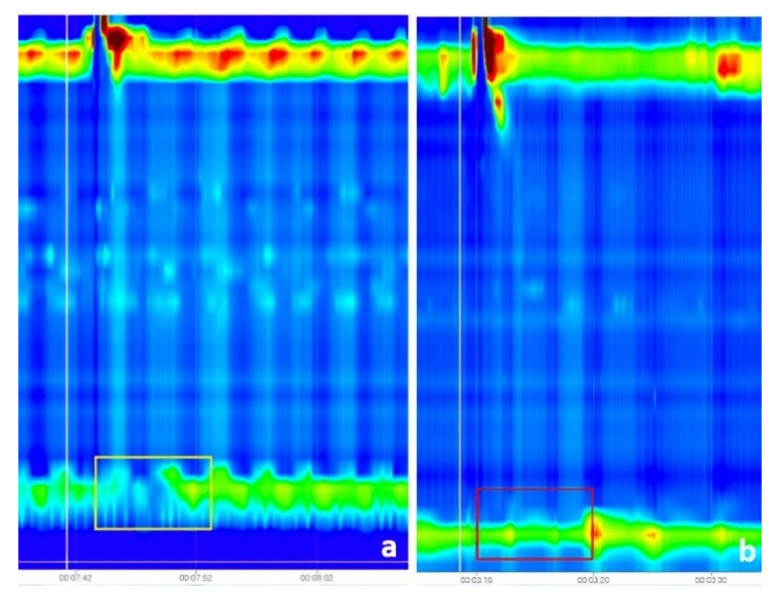
(**a**) Swallow with failed peristalsis and correct relaxation of lower esophageal sphincter (LES) (the color change was determined by a pressure drop, and the measured IRP was normal—the region of interest is marked by the yellow rectangle); (**b**) Swallow with failed peristalsis and absence of LES relaxation (the color remained constant, and the measured IRP was higher than the cut-off—the region of interest is marked by the red rectangle).

The second dataset contained labeled images regarding the IRP. It consisted of 1079 images from which 140 were images representing normal IRP and 939 were images representing IRP higher than the cut-off. The mean age of patients included in this dataset was 50.3 ± 17.5 years old, and 52.3% were males. Images for normal IRP were obtained from 14 patients: 6 with normal esophageal HRM, 3 with ineffective esophageal motility, 3 with absent contractility, 1 patient with fragmented peristalsis, and 1 with distal esophageal spasm. Images for higher than cut-off IRP were from 4 patients with esophago-gastric outflow obstruction and 90 patients with achalasia. All images were wet swallows without any added markings, except for the vertical white line (placed during the recording) for the test swallow. The images were saved using the software feature, which creates images representing 60 s of the recording, visible at the time on the screen. For analysis purposes, when images were saved, we made sure that the marking for the wet swallow was close to the middle of the image. Figure 1 and Figure 3 show examples for both normal and high IRP. Normally, after swallowing, the LES pressure drops for few seconds. The parameter that evaluates the swallow induced LES relaxation is the IRP, measured in the 10 s after swallowing (Figure 1 and Figure 3 in the yellow rectangle). In the case of a high IRP, there is little or no change in LES pressure after swallowing (Figure 1 and Figure 3 in the red rectangle).

The IRP is measured in the first 10 s after the initiation of the swallow (identified based on the white vertical line) and is compared with LES resting pressure (the pressure of LES when the patient is not swallowing). In cases of a normal IRP the pressure is very low (meaning that the sphincter relaxes correctly), while in the case of a high IRP, the change in pressure during swallowing is less important, sometimes unchanged compared to the resting pressure.

The objective of this article was to prepare the steps for automatizing the Chicago Classification algorithm. There were two steps in preparing the solution. One was to filter out the images in cases where the probe was positioned wrongly (probe positioning failure). The output of the first step would be the input for the second step. In the second step, we used only the images containing correct probe positioning and trained a model to classify the IRP parameter as normal or higher than the cut-off. The two steps are essential for being able to automatize the Chicago Classification with good results. If for the first task we needed both correct and probe positioning failure images, for the second task we required only images obtained with the correct probe positioning. In a real-life scenario, the first step will automatically exclude the images obtained during probe positioning failure. However, for our preliminary experiments regarding IRP classification, we prepared a different dataset that does not contain erroneous images.

### 2.2. Preprocessing of the Esophageal Pressure Topography Maps

The raw image (the 60 s image) contains more information than necessary for training an artificial neural network, which is considered as noise. As mentioned above, while creating the dataset, we made sure that the marking for the wet swallow was close to the middle of the image. To remove the noise from the raw images, we cropped them using the following rule: upper, lower, and right limits were the image borders, and the left border was represented by the white vertical line before each test swallow. Images representing 20–30 s of the recording resulted, which were sufficient to analyze the IRP, as IRP is measured in the first 10 s after the beginning of a wet swallow [3]. 

**Figure 4:**
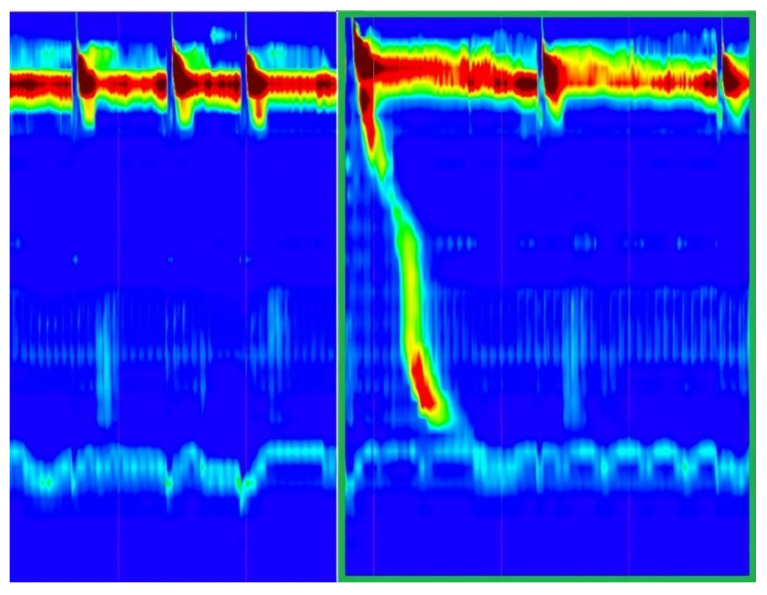
Raw image with the highlighted region of interest (green rectangle).

**Figure 5:**
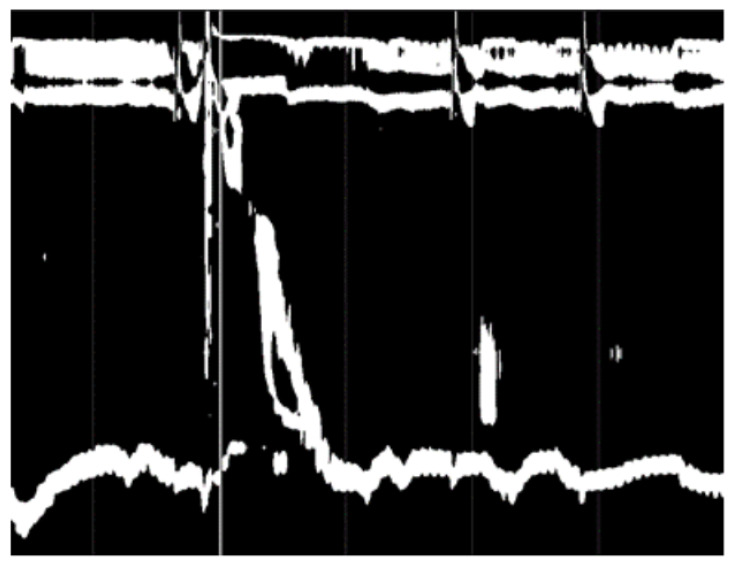
Binarized image.

**Figure 6:**
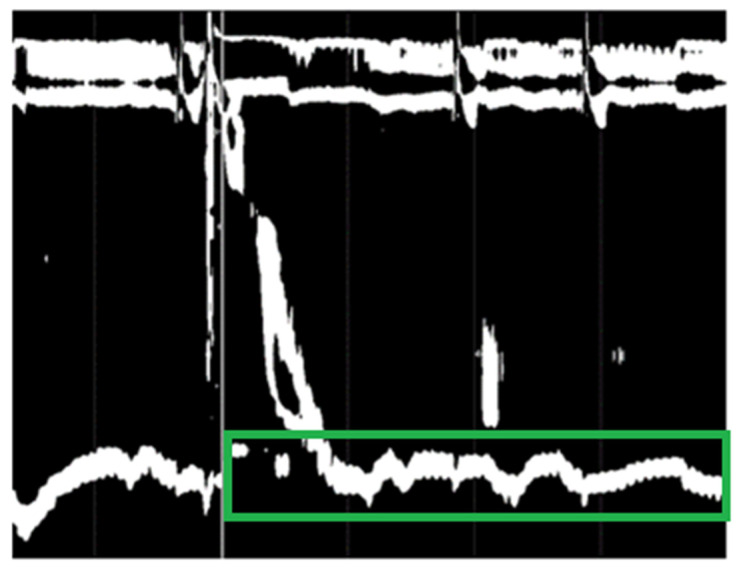
Binarized image with highlighted region of interest for IRP (green rectangle).

To automatically find the region of interest highlighted by the green rectangle in Figure 4, we started by eliminating the grey margins from the top, bottom, and left. For finding the white vertical line marking the wet swallow, we binarized the image (see Figure 5). In the next step, to find the vertical white line, we counted the white pixels along the *y*-axis (histogram of white pixels) and selected the index of the maximum pixel count, which is exactly the *x*-axis value that we wanted to find. To find the region of interest for the IRP classification, using the binarized image, in a bottom-up direction we found the first white pixel (white pixel with the maximum *y*-axis value) and then we cropped the image starting from the *y*-axis value of the found pixel using a rectangle of 100-pixel height (see Figure 6). Because the convolutional neural network (CNN) that we used for classification has an input shape of 299 × 299 × 3 and it works with values between −1 and 1, all of the images were rescaled and normalized to have values in the [−1, 1] interval. The whole dataset was split into three parts, one part for training, which contained 70% of the data (657 swallows with high IRP, 98 swallows with normal IRP), one for testing, containing 15% of the dataset (140 swallows with high IRP, and 21 swallows with normal IRP), and one for validation (remaining 15% of the dataset). 

To obtain the final model the CNN model must be trained multiple times (with the training dataset) while obtaining intermediate feedback about the quality of the model by using the test dataset. The intermediate feedback is used for improving the model during the training process. After finalizing the model, the validation dataset is used for the results validation. Having three different datasets will guarantee that the validation set was never seen by the model and in this way accurate evaluation scores can be obtained. The training set is the largest part of the dataset, reserved for training the model. The test set is used during the training to get a sense of how well the model assesses new images, that were not previously seen by the model. During training, it is common to report metrics continually after each training epoch such as the validation loss. Because the test set is heavily used in model creation and training, it is important to hold back a completely separate set of data. We ran evaluation metrics on the validation set at the very end of the research, to check how well the model would perform in real life.

### 2.3. Transfer Learning

Training a CNN from scratch would require thousands of images, but it is very difficult to obtain such a large amount of labeled medical images. An efficient way to solve the problem of small data is to use transfer learning [7]. In transfer learning, as a starting point for solving the problem of HRM image classification, another model was used that was trained for another classification task, for which much more labeled data was available. In our solution, we used the InceptionV3 CNN model [8] which was pre-trained on the ImageNet dataset [9]. The ImageNet dataset contains approximately 1 million images and 1000 classes. 

## 3. Results

### 3.1. Solution Pipeline

The first step of the solution pipeline was the preprocessing step. The preprocessing algorithm contained the following steps:

1. Delete the margins of the image; every image has a 15 pixels top margin, 120 pixels left margin, and a 30 pixels bottom margin which should be removed.
2. Binarize the image.
3. Calculate the histogram of white pixels for each *x*-axis position based on the binarized image created previously.
4. Find the maximum value of the previously calculated histogram. The *x*-axis position of the founded maximum value will be the *x*-axis position of the white vertical line.
5. Crop the original image (the colored image without the margins) starting from the previously found *x*-axis position until the end of the image.

**Figure 7:**
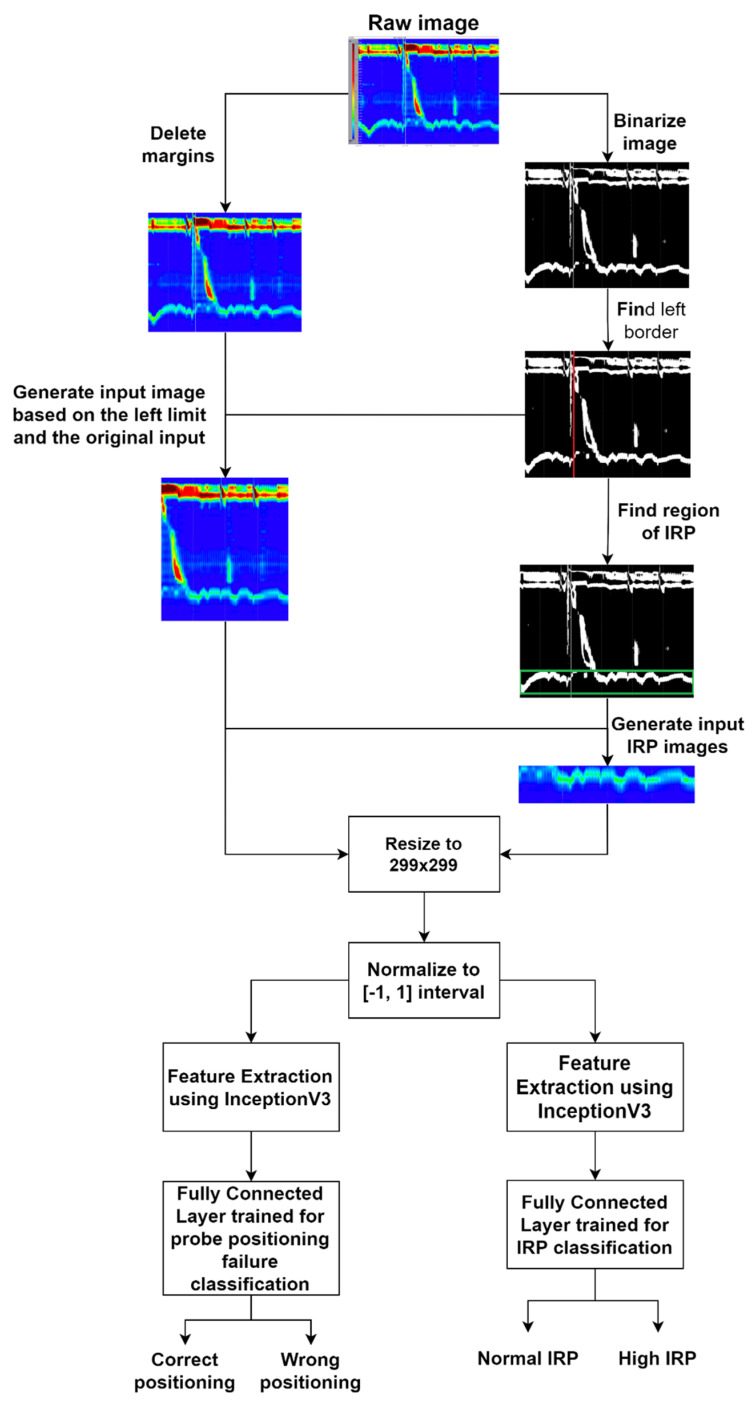
Solution pipeline.

The raw input image had some generated margins which could be considered to be noise. In the first step (see Figure 7) we deleted the noise by removing the top, left, and bottom margins. In the next step, we binarized the image using a threshold of 128 per pixel. In this way the white vertical line delimiting the wet swallow became more visible. In the next step, we found the *x*-axis position of the vertical white line by counting the number of white pixels along the *y*-axis for each position on the *x*-axis and choosing the position of the maximum count. In the next image pre-processing step, we used the previously found *x* position to crop the original image, finding exactly the part of the image that represented a single wet swallow. This image would be the input for the probe positioning failure detection CNN model and based on this image and the binarized image the part of the image which represented the IRP for a single wet swallow was found. This IRP image would be the input for the IRP classification CNN model. 

After pre-processing the raw image, we resized it to a dimension of 299 × 299, because the InceptionV3 model accepts an input image of this size. In the next step, we normalized all the pixel values to a range of [−1, 1], and the resulted matrix was fed to the feature extraction part. For the feature extraction step, we used the InceptionV3 CNN model, without the final classification layer, and pre-trained on the ImageNet dataset, in this way leveraging the power of transfer learning and overcoming the small data problem. We built two separate models, one for IRP classification and one for probe positioning failure detection. Both models used InceptionV3 as a feature extractor. In the final part of the models, we added a custom classification layer in which a global average layer [10] with a dropout of 20% was used, thus avoiding overfitting problems, and a final fully connected layer containing two neurons in both cases, because for both models there are two available outputs/classes. The model was trained using the Adam optimizer [11], with a batch size of 32 images and the data was shuffled in every epoch. 

### 3.2. Metrics

We employed several assessment criteria to conduct a thorough review of the solution:

- Accuracy: The number of correct classifications compared to the total number of examples.
- Precision: The ratio of the correctly classified positives to the total number of positive classifications.
- Recall: The ratio of the correctly classified positives to the total number of positives from the dataset.
- F1-Score: The harmonic mean of Precision and Recall.
- Confusion Matrix: A confusion matrix is a summary of prediction results on a classification problem. The number of correct and incorrect predictions are summarized with count values and broken down by each class. The confusion matrix shows how the classification model is confused when it makes predictions.

To correctly calculate these metrics, it is important to mention that in the case of the probe position failure detection, the positive class was represented by the correct positioning of the catheter, and in the case of the IRP classification problem, the positive class was represented by the normal IRP class.

### 3.3. IRP Classification Results

After preprocessing the whole dataset of images and finding the region of interest for IRP in each image, we trained our CNN model to classify normal or high IRP images. The results of the trained neural network were very promising, and the evaluation scores are presented in Table 1. 

**Figure 8:**
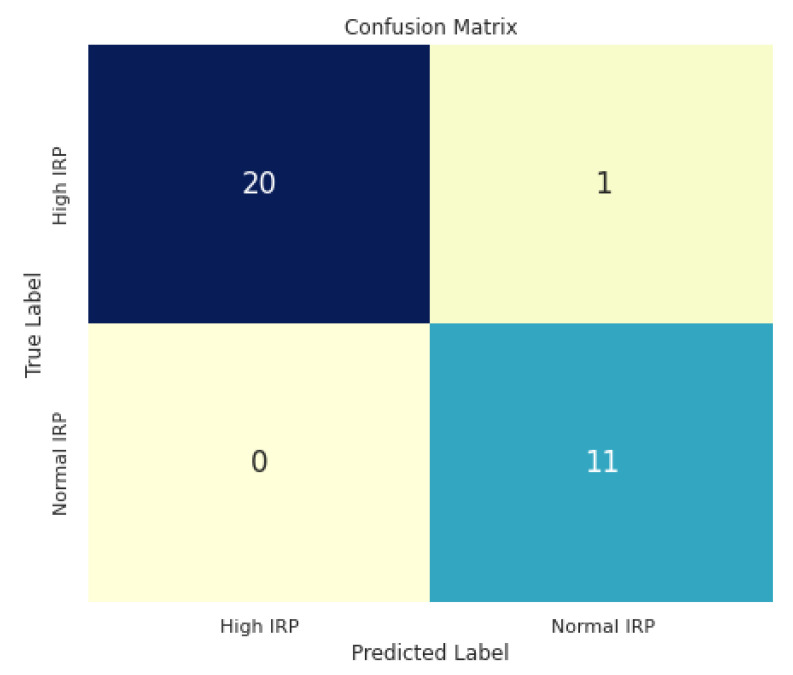
IRP classification confusion matrix.

**Figure 9:**
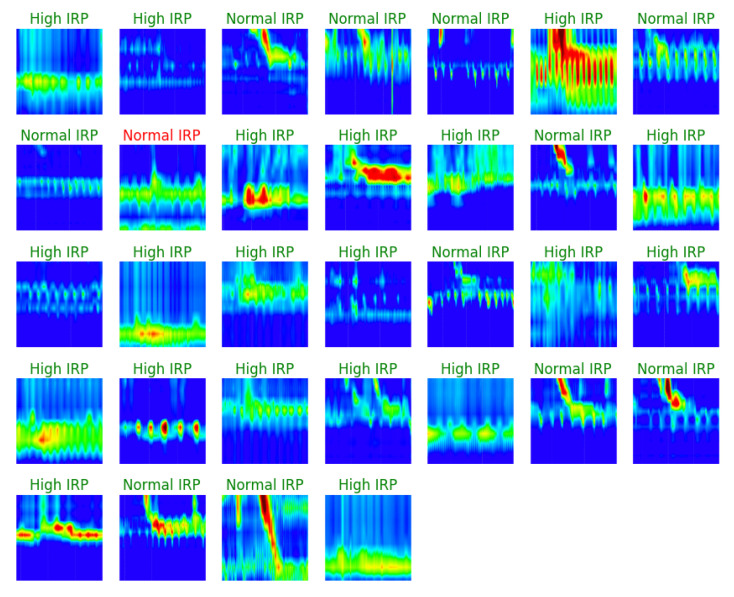
IRP classification—validation set examples with predicted labels.

The confusion matrix that was achieved on the validation set can be seen in Figure 8. Only one out of 32 images was incorrectly classified. In Figure 9 some examples from the validation set and the predicted label for them are presented. The correct labels were marked with green and the red color means that the image was miss-classified by the CNN model.

### 3.4. Probe Positioning Failure Classification Results

**Figure 10:**
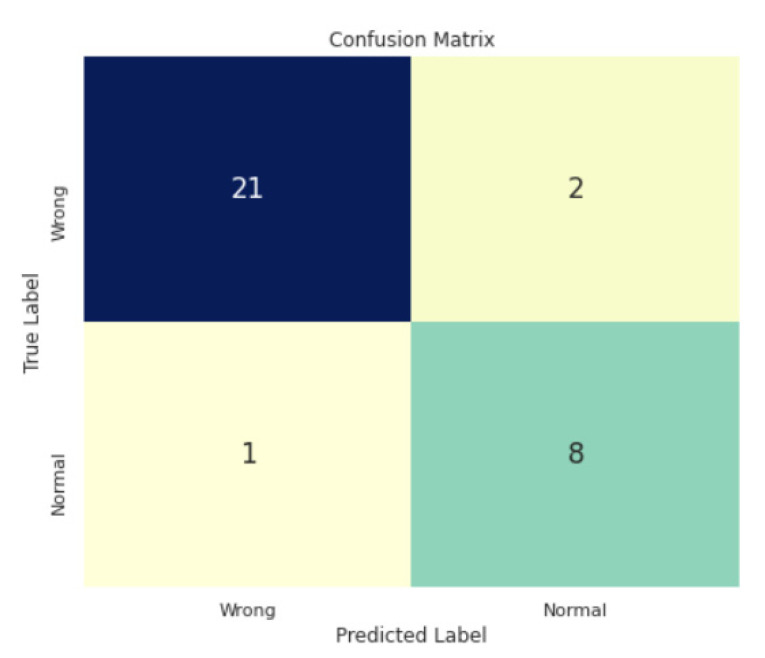
Probe positioning failure detection confusion matrix.

After passing the images through the pipeline presented in the previous section and after training our CNN model, the confusion matrix presented in Figure 10 was obtained. Only three out of 32 images were incorrectly classified by the model and the metrics are presented in Table 2. 

## 4. Discussion

Medicine has already benefited from the use of Artificial Intelligence (AI) and machine learning. Two recent reviews analyzed the applications of AI and machine learning in gastroenterology [12,13]. AI was used in polyp detection during colonoscopy [14], celiac disease diagnosis [15], predicting mortality in variceal bleeding [16], or prediction of liver fibrosis in hepatitis C virus infection [17]. Other uses of AI were used in the characterization of colorectal lesions [18], or the measurement of baseline impedance from pH-impedance studies [19]. 

Searching the literature, only a few articles tackle the problem of esophageal motility diagnosis or the automation of the Chicago Classification algorithm. Most of the studies analyzed only pharyngeal changes and swallowing patterns [20,21,22,23,24] and focused on changes at the level of upper esophageal sphincter, and did not analyze EMD or IRP; therefore, they cannot be compared to our study. Hoffman et al. [20] found a solution for classifying swallows as safe, penetration, or aspiration. The authors managed to train a Multilayer Perceptron with an accuracy of 89.4% on data extracted manually from the images by the specialist. The disadvantage of this solution is that they did not work on the raw images, but on manually extracted data, so the solution is not fully automated, as it still requires the input of a specialist. 

Mielens et al. [21] compared multiple models in order to identify disordered swallowing of the upper esophageal sphincter. To identify abnormal swallowing, a variety of classification methods, including artificial neural networks (ANNs), multilayer perceptron (MLP), learning vector quantization (LVQ), and support vector machines (SVM) were investigated. All methods produced high average classification accuracies, with MLP, SVM, and LVQ achieving accuracies of 96.44%, 91.03%, and 85.39%, respectively [21]. Once again, all these articles are referring to the pharyngeal time of swallowing, and not esophageal or LES changes. None of these articles considered IRP evaluation or classification.

To our knowledge, only three studies [25,26,27] tried to develop systems to diagnose EMD. One interesting paper comes from Frigo and coworkers [25], who proposed a physio-mechanical model to represent esophageal movement and the propagation of the peristaltic pressure wave. The challenge of finding the relevant model parameters from HRM data was therefore transformed into an optimization problem, where the cost function described the difference between HRM data and model outputs, and the goal was to reduce this discrepancy. The authors included 226 recordings of both healthy and pathological subjects. First, the model parameters were defined. Motility disorders influence the physio-mechanical properties of different regions along the esophagus, and therefore, determine a significant variation in a specific parameter (for example, a parameter which characterizes the functionality of the LES). The statistical relationships between the parameters were determined to describe the esophageal function in different groups of subjects. Based on these parameters, a preliminary database was created. Afterwards, an automated statistical algorithm was developed to compare the parameters observed in a subject with the model parameters from the database. The correct diagnosis was achieved in 86% of cases using this algorithm [25]. In this paper, the problem of normal or high IRP was included in the physio-mechanical model. In patients with non-relaxing LES (equivalent of a high IRP), significantly lower values of Δ parameter were observed, compared to healthy controls [25]. In comparison with the expert system described above, a similar problem was tackled, the problem of IRP classification, but in our case the solution was fully automatic, the features were automatically extracted by the CNN with no human intervention, obtaining an accuracy of over 96%. We consider this better suited compared with a physio-mechanical model prepared by human experts.

Kou et al. [26] proposed an unsupervised deep learning solution to automatically identify new features and attributes that could be used in esophageal motility diagnosis, starting with swallow-level raw data. They proposed and trained a variational auto-encoder to group images in six main categories based on the swallow type (normal, weak, failed, fragmented, premature, hypercontraction) and three categories based on the pressurization type (normal, compartmental, panesophageal pressurization). The authors used a dataset of more than 30,000 images of raw swallows. After grouping the images, they used the linear discriminant algorithm and then the principal-component analysis to reduce the dimensionality of the data and to find the most important attributes, based on which the grouping was done [26]. In this article [26], the IRP was not considered. Because IRP is a very important parameter when interpreting esophageal HRM, we chose to start with its classification in normal versus high, and a future project will address the swallow pattern. 

Jell et al. evaluated the feasibility of autonomous analysis of ambulatory long-term HRM using an AI-based system [27]. This technique is used when a temporary EMD is suspected. During the 24 h recording of esophageal motility, around 900 swallows appear, and an enormous amount of data is generated. In the study, 40 patients with suspected EMD were used for the training and evaluation of a supervised machine learning algorithm for automated swallow detection and classification. Nevertheless, the HRM results were previously manually tagged. In the end, the evaluation time of the entire recording was reduced from 3 days to 11 min, for automated swallow detection and clustering, plus another 10–20 min for evaluation. Furthermore, the AI-based system was able to reveal new and relevant information for the patient’s therapeutic recommendation [27].

In contrast to the solutions presented above, our final goal is to fully automate the Chicago Classification algorithm, meaning that the final solution would be capable of classifying EMD based on raw images, with no input from physicians. This work is the first part of the final algorithm, and is focused on the two most important steps before applying the Chicago algorithm, namely probe positioning failure detection, which would make the interpretation of HRM recording impossible, and IRP classification. IRP is one of the main parameters in the Chicago Classification and in the algorithm. Therefore, our first objective was to determine whether the IRP is normal or higher than the cut-off, based solely on raw images. The second objective was to identify if the catheter was positioned correctly, allowing swallow interpretation. The innovation of our approach is supported by both the few references quoted and two recent reviews [12,13] on applications of AI to gastroenterology failing to consider its potential to improve diagnosis of EMD.

For probe positioning failure detection, our dataset was very unbalanced, which is normal, because the specialists always try to position the catheter correctly. The recall results were smaller compared with the accuracy. This shows the effects of not having enough examples for the wrong positioning situation. We cannot apply traditional data augmentation techniques to increase the number of wrong positioning examples, because the position, the scale, and the angles are important, and obtaining these images manually would be hard because it would require positioning the catheter wrongly in a conscious manner, which would be unethical and could hurt the patient. In the future, we will try to obtain more images from other hospitals, in this way improving the performance of the probe positioning failure classifier.

## 5. Conclusions

In this article we presented a solution for detecting the catheter positioning failure in esophageal HRM and we also trained a model to classify IRP as normal or high. In the first part of the article, we defined HRM and presented the steps of the procedure, the resulted images, and the disorders that can be detected with it. In the second part we described the preprocessing steps that were necessary to prepare the input data for the CNN models and to find the region of interest for the IRP classification problem. Then we presented the solution pipeline which was used to setup and train the models using the preprocessed images. We used two InceptionV3 deep learning models to classify the images as correct or failure in catheter positioning and to determine the exact class of the IRP. To overcome the problem of small training data, as a starting point for feature extraction we used pretrained InceptionV3 models (pretrained on the ImageNet dataset) and changed the last fully connected part to match the size of our specific problem.

In the last part of the article, we presented the experimental results. These results were quite impressive, achieving an accuracy over 90% and an F1-score over 84% in both cases. Compared to other articles/attempts to automatically detect IRP higher than the cut-off, the advantage of our solution is that it is fully automatic, the features used for classification were automatically obtained by the InceptionV3 CNN model, and the classification was made based on these features with no human intervention. Furthermore, using the pre-trained InceptionV3 model we obtained even higher evaluation scores than in other articles, where the features and attributes were prepared manually by human experts.

This work is just the first step in fully automating the Chicago Classification (version 3.0) algorithm, in this way assisting hospitals and doctors in their daily workplace and reducing costs and time wasted on repetitive tasks.

## Figures and Tables

**Table 1:** Evaluation metrics for IRP classification.

Class	Precision	Recall	F1-Score
IRP higher than cut-off	95%	100%	98%
Normal IRP	100%	92%	96%
Overall Accuracy			97%

**Table 2:** Evaluation metrics for probe positioning failure classification.

Class	Precision	Recall	F1-Score
Wrong	91%	95%	93%
Normal	89%	80%	84%
Overall Accuracy			91%

## Data Availability

Data available on request due to restrictions eg privacy or ethical. The data are not publicly available due to the sensitive nature of medical data.
